# Tail‐Cuff Technique and Its Influence on Central Blood Pressure in the Mouse

**DOI:** 10.1161/JAHA.116.005204

**Published:** 2017-06-27

**Authors:** Elena Wilde, Aisah A. Aubdool, Pratish Thakore, Lineu Baldissera, Khadija M. Alawi, Julie Keeble, Manasi Nandi, Susan D. Brain

**Affiliations:** ^1^ Vascular Biology and Inflammation Section BHF Cardiovascular Centre of Research Excellence Cardiovascular Division King's College London London United Kingdom; ^2^ Pharmaceutical Sciences Division King's College London London United Kingdom

**Keywords:** high blood pressure, hypertension, mouse, noninvasive blood pressure measurement, stress, tail‐cuff, telemetry, Animal Models of Human Disease

## Abstract

**Background:**

Reliable measurement of blood pressure in conscious mice is essential in cardiovascular research. Telemetry, the “gold‐standard” technique, is invasive and expensive and therefore tail‐cuff, a noninvasive alternative, is widely used. However, tail‐cuff requires handling and restraint during measurement, which may cause stress affecting blood pressure and undermining reliability of the results.

**Methods and Results:**

C57Bl/6J mice were implanted with radio‐telemetry probes to investigate the effects of the steps of the tail‐cuff technique on central blood pressure, heart rate, and temperature. This included comparison of handling techniques, operator's sex, habituation, and influence of hypertension induced by angiotensin II. Direct comparison of measurements obtained by telemetry and tail‐cuff were made in the same mouse. The results revealed significant increases in central blood pressure, heart rate, and core body temperature from baseline following handling interventions without significant difference among the different handling technique, habituation, or sex of the investigator. Restraint induced the largest and sustained increase in cardiovascular parameters and temperature. The tail‐cuff readings significantly underestimated those from simultaneous telemetry recordings; however, “nonsimultaneous” telemetry, obtained in undisturbed mice, were similar to tail‐cuff readings obtained in undisturbed mice on the same day.

**Conclusions:**

This study reveals that the tail‐cuff technique underestimates the core blood pressure changes that occur simultaneously during the restraint and measurement phases. However, the measurements between the 2 techniques are similar when tail‐cuff readings are compared with telemetry readings in the nondisturbed mice. The differences between the simultaneous recordings by the 2 techniques should be recognized by researchers.

**Clinical Perspective**

**What Is New?**

- This study provides a step‐by‐step analysis of the components of the tail‐cuff technique and their effect on hemodynamics and core body temperature.
- It reveals that restraint induces a stress response that is neither ameliorated by habituation nor different handling techniques.
- The detailed comparison of the noninvasive tail‐cuff and the invasive telemetry blood pressure measurement techniques reveal that the tail‐cuff systematically underestimated central blood pressure as recorded by telemetry simultaneously in the same mouse; however, the tail‐cuff recordings were similar to those obtained by telemetry in undisturbed mice.

**What Are the Clinical Implications?**

- The tail‐cuff technique is commonly used to determine physiological and pathological blood pressure measurements in murine models of human disease.
- The results aid interpretation of studies that use the tail‐cuff technique and provide evidence where measurements with this technique may be misinterpreted, especially in terms of central blood pressure analysis.
- These findings could have a significant impact on the use of the tail‐cuff technique going forward, providing evidence of the need to ensure rigorous standards in some (eg, temperature), but not all of the specific factors involved.

## Introduction

Cardiovascular research relies heavily on accurate and reliable blood pressure measurements in conscious mice. There are 2 main techniques available: radio‐telemetry (referred to here as telemetry) and the tail‐cuff technique. An important challenge imposed by the conscious state is that stress can be generated by experimental procedures. The term “stress” is used to define a range of conditions that may threaten an organism or are perceived as such.^1^, ^2^, ^3^ The physiology of the stress response encompasses the entire body and therefore should be an important consideration in experimental protocols and data interpretation.

The measurement of blood pressure in mice by pre‐implanted telemetry probes allows continuous measurement of unrestrained animals in their home environment.^4^ This removes the stress factor associated with human interaction or restraint. By comparison, the tail‐cuff is a noninvasive technique that is more economical and efficient, but it requires handling, warming, and restraint of the animals. This generates stress and has been suggested to be a major limitation of the tail‐cuff technique.^5^

Although telemetry is a direct measurement of central blood pressure (from the aortic arch as in our case) and is considered the “gold standard” technique,^5^ it requires the mice to undergo major surgery for the implantation of the telemetry probes and subsequent recovery. The probe implantation involves permanent ligation of an artery, most commonly the left carotid in mice, which is a significant perturbation to the circulation.^6^ The weight of the probe can compromise animal welfare,^7^ as well as the requirement for single housing for the purposes of data collection.^8^, ^9^ Although telemetry systems compatible with group housing are becoming available for mice, a relatively high cost and requirement for major surgery remain important limiting factors. The tail‐cuff technique does not have these limitations and is widely considered to be acceptable and recommended for certain applications such as phenotypic screening and studies that involve large numbers of mice.^5^, ^10^ Despite being associated with stress, some consider the tail‐cuff technique to be more acceptable for the 3Rs’ term of “refinement” because it is performed without the need for anesthesia or surgery. Both techniques are widely used in the mouse. Therefore, it is important to understand the impact of the steps involved in the tail‐cuff procedure and the scope for the refinement of the technique.

A number of studies have investigated the effect of handling using several stress‐related biomarkers, including changes in blood pressure, heart rate, core temperature, stress hormones, immune responses, and behavior.^11^, ^12^, ^13^ Although handling is accepted as stressful to mice, there is no total agreement as to whether mice can be habituated to handling with a meaningful reduction in the stress response.^11^, ^14^

Handling is made up of many variables including the specific technique of how the mouse is captured and other factors such as the handler and the environment. Moreover, differences in sensory adaptations between humans and mice render the scientist unaware of many environmental variables, such as auditory and olfactory stimuli, that can affect experimental conditions.^15^ Interestingly, Sorge et al^12^ found that male, rather than female, operators/researchers are potentially more stressful to mice. They provided evidence that the presence of human male, but not female, scent in the room induced a significant increase in mouse stress hormones. Hurst and West revealed that certain handling techniques can result in less anxiety in the mouse.^13^ Anxiety is a complementary concept of the stress response characterized by involvement of higher behavioral responses such as emotions.^2^ Although the effect of different handling techniques on the anxiety level in the mouse is clear, the effect of the different handling techniques on blood pressure has never been quantified.

The technologies for both telemetry and tail‐cuff blood pressure monitoring have evolved since these were first described for use in rats.^5^, ^16^, ^17^, ^18^ The need to handle, restrain, and warm the animals has remained a necessity for the tail‐cuff technique. These limitations of the tail‐cuff technique are established.^19^, ^20^ However, a systematic and comprehensive evaluation of these stress‐inducing factors and their effect on hemodynamics in mice is lacking. It is believed that the mice habituate to the tail‐cuff procedure following repeated exposure and there is a common practice to “train” mice before starting an experiment.^10^, ^21^, ^22^ However, there is evidence that this does not happen.^23^, ^24^ Moreover, the tail‐cuff technique has been used to model stress that was defined by measuring heart rate.^23^

A further important consideration is the anatomical position from which the measurements are taken. Telemetry devices take measurements directly from inside a major blood vessel, commonly the aortic arch or abdominal aorta in the mouse. The currently used tail‐cuff sensor technologies rely on detection of either flow or pulse following the occlusion of the tail artery, thus measuring the blood pressure at the peripheral site. Volume Pressure Recording (VPR) sensor technology for tail‐cuff, presently one of the most widely used, was validated by Feng et al.^10^ It is commonly cited that they found negligible difference between telemetry and the VPR systems,^25^, ^26^ despite an apparent difference in individual recordings or lack of consistent results across the whole range of blood pressure recordings.

In this study, we explore how different handling techniques, the handler's sex, and habituation affect the mouse, in terms of central blood pressure and heart rate as measured by telemetry. We then progressed to examine the impact of handling, restraint, and warming on central blood pressure and core body temperature. Finally, we compared the central blood pressure readings obtained by the tail‐cuff and telemetry in the same mice after induction of raised blood pressure in a commonly used angiotensin II (AngII) hypertension model.^27^

## Methods

### Animals

In vivo experiments were performed according to the UK Home Office Animals Scientific Procedures Act 1986 and Amendment Regulations 2012 and approved by King's College London Animal Welfare and Ethical Review Body. Animal Research: Reporting of In Vivo Experiments guidelines were used for reporting the procedures.^28^ Male and female C57Bl/6J mice aged 13 to 15 weeks were used. The numbers of mice used in each experiment were as follows: n=6, all males (the effect of handling), n=3 all males (the effect of handler's sex), and n=4 per group, males and females (for the effect of heating and other handling interventions on hemodynamics and core body temperature). The mice had free access to food and water and were maintained in a climatically controlled environment (22±2°C) with humidity (50±10%) under filtered positive pressure ventilation on a 12/12 hours dark/light cycle beginning at 07:00 GMT. Mice were housed in opaque polypropylene cages, size 45×28×13 cm (North Kent Plastics, UK), with wood chip bedding, paper nesting material, and cardboard enrichment tubes. All mice were singly housed following the implantation of the telemetry probes.

### Measurement of Blood Pressure by Tail‐Cuff Plethysmography

The CODA 8 noninvasive blood pressure acquisition system for mice (Kent Scientific, Torrington, CT) was used for all tail‐cuff measurements. This system uses VPR to detect blood pressure based on volume changes in the tail. CODA system was factory calibrated and standard settings and recommendations were used^21^ as follows. Patency of the occlusion and VPR cuffs was checked routinely before the start of the experiments. The blood pressure measurement experiments were conducted in a designated quiet area (22±2°C), where mice acclimatized for a 1‐hour period before experiments began. Thereafter, the mice were subjected to experimental protocols as detailed below. For all, mice were encouraged to walk into the restraint tubes and the tube end holders were adjusted to prevent excessive movement. The occlusion cuff was placed at the base of the tail and the VPR sensor cuff was placed adjacent to the occlusion cuff. Heating pads, supplied as part of the CODA 8 system, were preheated to 33 to 35°C. The mice were warmed for 5 minutes before and during blood pressure recordings. To measure blood pressure, the occlusion cuff is inflated to 250 mm Hg and deflated over 20 s. The VPR sensor cuff detects changes in the tail volume as the blood returns to the tail during the occlusion cuff deflation. The minimum volume change was set as 15 μL. Each recording session consisted of 15 to 25 inflation and deflation cycles per set, of which the first 5 cycles were “acclimation” cycles and were not used in the analysis, whereas the following cycles were used. Mice were habituated for at least 5 consecutive days before baseline blood pressure measurements.

### Surgical Procedures for Implantation of Telemetry Probes

All surgical procedures described below were conducted using aseptic techniques under isoflurane anesthesia (2%, Abbott Laboratories, UK) in 2 L/min O_2_. Buprenorphine was administered perioperatively (50 μg/kg, i.m., Vetergesic; Sogeval UK Ltd) for pain relief. The abdomen was shaved and wiped with surgical iodine. For blood pressure, heart rate, and activity measurements the telemetry probe (PA‐C10; Data Science International [DSI], St. Paul, MN) was inserted. During anesthesia and following abdominal incision, the catheter of the transducer was inserted in the left carotid artery and advanced towards the aortic arch. The catheter was secured using surgical braided silk (5.0, waxed sutures, Pearsalls Ltd, UK) and the transmitter was placed subcutaneously in the right flank. The abdominal wall and the skin incision were sutured separately using absorbable sutures (Vicryl 5.0; Ethicon, Johnson & Johnson).^29^ For measurement of temperature, the transmitter (TA10TA‐F10; DSI) was inserted. For this, a ventral incision was made on the abdominal wall and irrigated with sterile saline (0.9% saline; sodium chloride, pyrogen free) to facilitate the insertion of the radiotelemetry transmitter. The outer wound was closed with absorbable sutures as above.^30^ All mice were singly housed and following a 10‐day recovery period, either the central blood pressure and heart rate, or temperature were recorded continuously at 1‐ to 10‐minute scheduled intervals.

### Simultaneous and Nonsimultaneous Blood Pressure Telemetry and Tail‐Cuff Recording

Telemetry blood pressure was acquired at the same time as tail‐cuff recordings in the same mouse for simultaneous comparison. This was achieved by placing the telemetry receiver pad adjacent to the tail‐cuff device. Computer clocks on the telemetry and tail‐cuff systems were synchronized; 2‐ or 10‐s segments of telemetry recordings were acquired throughout the duration of the recording by tail‐cuff. To compare the recordings obtained simultaneously, individual readings were temporally aligned (approximate temporal resolution 2–5 s).

To compare nonsimultaneous recordings obtained by telemetry with the tail‐cuff readings, when the mice were neither restrained nor otherwise stressed, we used blood pressure recordings obtained on the same day for each mouse before handling or tail‐cuff measurements. Typically, a 15‐minute period was chosen 0.5 to 2 hours before the mice were disturbed. One‐minute averages for blood pressure recordings by the telemetry system during that period were compared with readings obtained by the tail‐cuff technique on the same day.

Having observed no significant differences in blood pressure between male and female mice as measured by both techniques at baseline or when the 2 techniques of measuring blood pressure were compared, we combined the data for both data sets for further analysis.

### Effect of Different Handling Techniques on Acute Blood Pressure During the Tail‐Cuff Protocol

The following 3 common mouse‐handling techniques were defined and tested based on findings by Hurst and West^13^ and our own observations. These were named “tube,” “tail,” and “tail cup,” as described below. For the “tube” technique, the environmental enrichment tube (normally present in the home cage) was used. The mice were lifted from the home cage in these tubes and transferred into the tail‐cuff restraint tube, with minimal handling. For the “tail” technique, the mice were picked up by the base of the tail, then supported on the back of the hand and moved to the platform before being transferred to the tail‐cuff restraint tube. For the “tail‐cup” technique, the animals were immobilized by the base of the tail and lifted up in the palm of the hand to the platform to be placed in the restraint tube.

Each mouse (total n=6) underwent each handling technique during the experiment in a semirandom manner. This was achieved by randomizing each mouse to a handling technique on the first test week so that the 3 handling techniques were tested in the same period, then again in the second week (ensuring the technique was different from the one used in the first week). In the third week the remaining handling technique for each mouse was used. Each test period was for 5 days, with a rest period (6 days) in between (Figure S1).

To study the acute effect of each handling technique on blood pressure and heart rate within the tail‐cuff protocol, it was arbitrarily subdivided into the following steps: “baseline” (the period before the animals were disturbed), handling (typically 10–30 s), placing the animal in the restraint tube (30–60 s), acclimatization in the restraint tube (typically 5 minutes), and tail‐cuff recording cycles. Telemetry was used to monitor blood pressure and heart rate during the experiment.

### Effect of Handler's Sex on Mouse Blood Pressure and Heart Rate During the Tail‐Cuff Protocol

Tail‐cuff protocols and telemetry recordings were carried out as described above. Each researcher handled 3 mice by their preferred technique (techniques used were recorded). Male (n=3) and female (n=4) researchers alternated to carry out the measurements on consecutive days.

### Effect of Heating and Handling Interventions Involved in the Tail‐Cuff Technique on Blood Pressure and Core Body Temperature

The following factors and steps are associated with obtaining tail‐cuff measurements: presence of the researcher in the room, moving the mouse in the cage next to the equipment, handling to place the mouse in the restraint tube, heating, and finally measuring blood pressure by the tail‐cuff (Figure S2). Telemetry was used to learn about the effect of these interventions on mouse central blood pressure, heart rate, and core temperature.

The following experimental procedures were performed typically on separate days repeated on at least 2 occasions for each animal. The researcher would enter and remain in the room for an ≈3‐minute period. The mouse in the cage was moved close to the platform next to the tail‐cuff machine and left for 15 minutes without handling. To test the effect of handling alone, the mice were picked up from the home cage and held in the hand for ≈30 s and then returned to the home cage for further recording over 15 minutes. For the effect of restraint without heating, the mice were placed in the restraint tube without preheating the tube or the underlying platform. To test the effect of restraint and heating, the mice were placed into the restraint tube preheated and maintained at 33 to 35°C. In both cases the restraint period lasted 15 minutes. The final step involved the tail‐cuff procedure. For this the mice were removed from the home cage, placed in the warmed restraint tube on the preheated platform as described above, and subjected to tail‐cuff recording after 5 minutes of acclimatization on the platform. Each recording session consisted of 15 inflation and deflation cycles of the cuff over the mouse's tail, with the first 5 cycles being used as “acclimatization” cycles and not used in the analysis. The telemetry data (temperature or cardiovascular) were collected continuously at 10‐s segments and presented as 1‐minute averages.

### AngII Murine Hypertension Model

The mice were trained to the tail‐cuff plethysmography protocol as described above and baseline blood pressure recording was obtained on 2 consecutive days. Thereafter, the animals were surgically implanted with osmotic minipumps (1002 Alzet osmotic minipump; Durect) containing AngII (Sigma) at a dose of 1.1 mg/kg per day or the vehicle saline (control) for 14 days, as previously described.^27^ Blood pressure measurements using telemetry were resumed. Tail‐cuff plethysmography was carried out on days 3, 5, 9, and 11 following minipump implantation and compared to simultaneous and nonsimultaneous recordings by telemetry.

### Statistical Analysis

Data in this article are expressed as mean±SEM. Statistical analysis was performed using GraphPad Prism 5.0 or IBM SPSS Statistics 22 software; *P*<0.05 was considered to represent a statistical significance. Statistical analysis was as described for each figure and typically included repeated measures‐ANOVA as most of the experiments used repeated measures in the same mouse. However, if the same parameter was measured in the same mouse on several days, only the average value was used for further analysis. To assess correlation and agreement between the 2 techniques to measure blood pressure, Bland‐Altman analysis was used to compare with data obtained by other groups.^10^ Pearson correlation was used to assess linear correlation between the 2 measuring techniques and regression analysis was used to support the correlation analysis in terms of what proportion of data can be explained by the fitted linear model. Correlation was considered strong for Pearson *r*>0.7 (or −0.7), medium for Pearson *r* between 0.7 and 0.5 (−0.7 and −0.5), weak for Pearson *r*<0.5 and 0.3 (−0.5 and −0.3), and negligible at <0.3 (−0.3).^31^

## Results

### Effect of Different Handling Techniques on Acute Blood Pressure During the Tail‐Cuff Protocol

**Figure 1:**
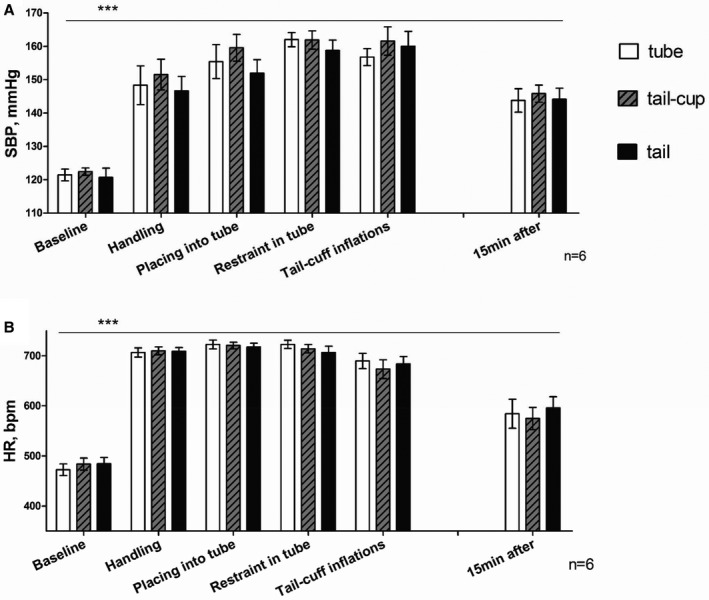
Hemodynamic responses to the stages of the tail‐cuff protocol depending on handling technique used. A, Changes in systolic blood pressure (SBP) and (B) heart rate (HR) during the stages of the tail‐cuff protocol as measured by telemetry. Values are mean±SEM for 6 mice over 5 recording sessions each. ANOVA for Latin‐Square design analysis (SPSS 22) showed there is no significant difference in SBP or HR between handling techniques at any stage of the protocol (*P*=0.694). However, each stage of the protocol is significantly different from baseline (***P<0.001). Values are mean±SEM for 6 mice. bpm indicates beats per minute.

To investigate the impact of the different handling techniques on hemodynamics at the point of handling and then during the tail‐cuff protocol, we have arbitrarily subdivided the tail‐cuff protocol into the following steps (Figure 1): *handling* (the animals are picked up from the cage by a specified technique), *placing into the tube*,* restraint in the tube* (acclimatization), *during tail‐cuff recording*, and at 15* *minutes after the mouse was returned to the home cage.

The 3 handling techniques did not differ (*P*>0.05) in the way that the blood pressure and heart rate (Figure 1) were affected; similar increases of systolic blood pressure and heart rate were observed for all the handling techniques versus baseline. The significantly elevated blood pressure and heart rate compared with baseline (*P*<0.001, Figure 1) were maintained throughout the protocol period during which the mouse was restrained and at least 15 minutes after the mouse was returned to the home cage.

### Effect of Handler's Sex on Mouse Blood Pressure and Heart Rate During the Tail‐Cuff Protocol

**Figure 2:**
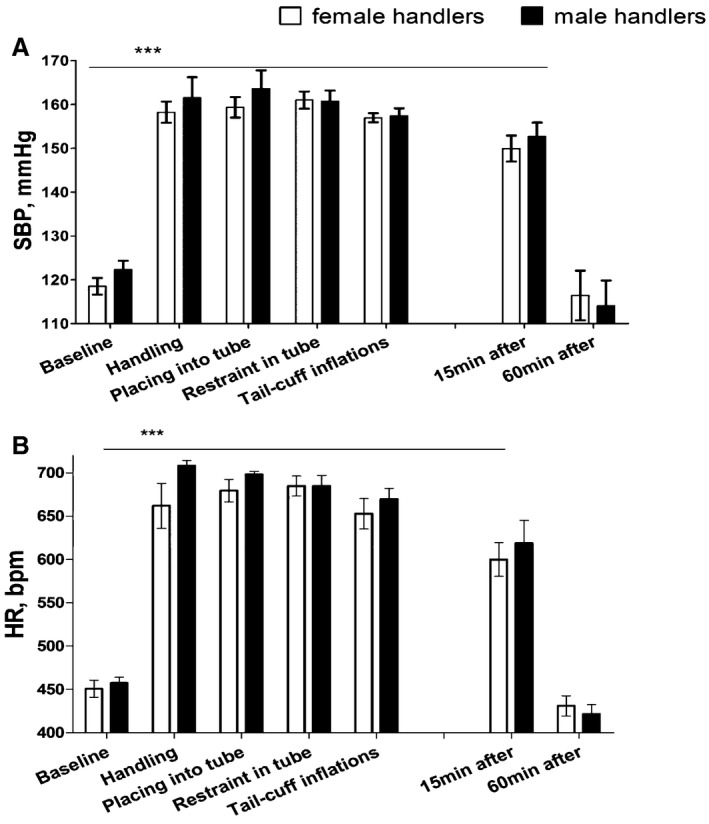
Hemodynamic changes in response to handling by male or female handlers during the stages of the tail‐cuff protocol. A, Changes in systolic blood pressure (SBP) and (B) heart rate (HR) during the stages of the tail‐cuff protocol as measured by telemetry. Values are mean±SEM for 3 mice over 7 recording sessions performed by 4 female and 3 male researchers on different days. Two‐way ANOVA showed there is no significant difference between male (n=3) or female (n=4) handlers at any stage of the protocol (*P*>0.05). However, each stage of the protocol is significantly different from baseline (***P<0.001), apart from the time point 60 minutes after the tail‐cuff protocol was finished (*P*>0.05). bpm indicates beats per minute.

The tail‐cuff protocol was arbitrarily separated into the sequential steps as described previously for the handling experiment. Blood pressure and heart rate were affected in a similar manner whether male or female researchers handled the mice throughout the tail‐cuff protocol (*P*>0.05) as shown in Figure 2. All stages of the protocol were significantly different from baseline (*P*<0.001), similar to the results of the previous experiment. Significant increases in blood pressure and heart rate compared with baseline were observed, which were maintained throughout the tail‐cuff protocol and up until 1 hour after the mice were returned to home cages (Figure 2). The decrease in blood pressure and heart rate following the tail‐cuff measurement was similar whether the mice were handled by male or female researchers.

### Effect of Restraint and Heating on Blood Pressure, Heart Rate, and Core Body Temperature

**Figure 3:**
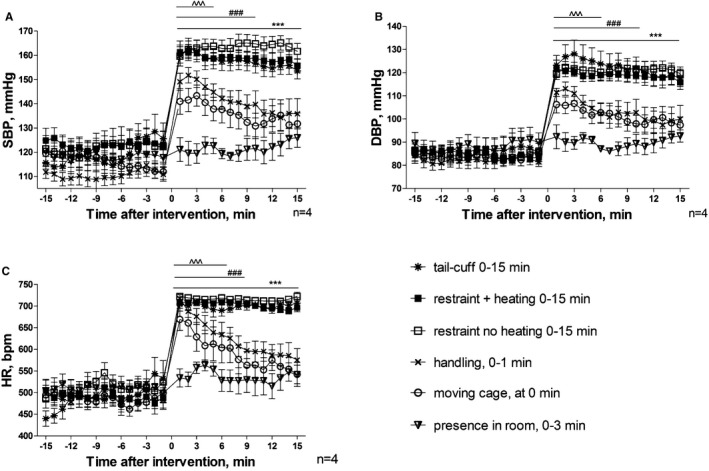
Hemodynamic responses to interventions associated with the tail‐cuff technique as determined by telemetry. A, Changes in systolic blood pressure (SBP). B, Changes in diastolic blood pressure (DBP). C, Changes in heart rate (HR). Interventions took place within the following periods: presence of the researcher in the room from between 0 and 3 minutes, cage was moved at 0 minute, handling took place between 0 and 1 minute, restraint with and without heating and tail‐cuff started at 0 and lasted until 15 minutes. Values are mean±SEM for 4 animals for each intervention, ^^^^^ *P*<0.001 moving cage, ^###^ *P*<0.001 handling, ***P<0.001 for restraint with and without heating and tail‐cuff intervention compared to baseline measurements using 2‐way‐repeated‐measures ANOVA and Bonferroni post‐hoc comparison test.

**Figure 4:**
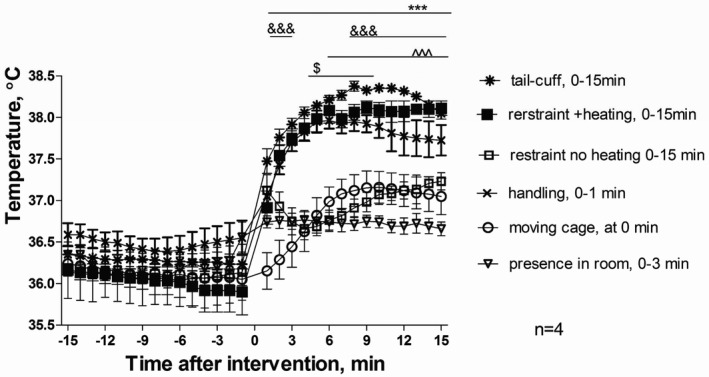
Core temperature changes in response to interventions associated with the tail‐cuff technique as determined by telemetry. Interventions took place within the following periods: presence of the researcher in the room was between 0 and 3 minutes, cage was moved at 0 minute, handling took place between 0 and 1 minute, restraint with and without heating and tail‐cuff started at 0 and lasted until 15 minutes. Values are mean±SEM for 4 animals, ^$^ *P*<0.05 for presence in the room, ^^^^^ *P*<0.001 moving cage, ^&&&^ *P*<0.001 restraint without heating, ***P<0.001 for handling, restraint with heating, and tail‐cuff intervention compared to baseline measurements using 2‐way‐repeated‐measures ANOVA and Bonferroni post‐hoc comparison test.

The following interventions were identified and extended from those described above for the tail‐cuff protocol: presence of the investigator in the room (0–3 minutes), moving the cage (at 0 minute), handling the animals (0–1 minute), restraint, heating to 33 to 35°C while in restraint, and performing the tail‐cuff recording itself (all 0–15 minutes). The effect on core body temperature, in addition to hemodynamics, was assessed using telemetry (Figures 3 and 4).

The presence of the investigator in the room did not cause significant perturbations in blood pressure; however, there was a trend of transient increase in heart rate and core body temperature observed during the 15‐minute recording period (Figures 3C and 4). All handling interventions, including moving the mouse in its home cage, significantly increased the blood pressure and heart rate (Figure 3) from the first minute (*P*<0.001). Maximal increases in blood pressure and heart rate were achieved when restraint was used. Warming to 33 to 35°C or tail‐cuff inflations did not further increase these parameters.

Cage movement across the room caused significant rise in blood pressure and heart rate immediately and until the eighth minute after the action was complete (*P*<0.001). The responses following handling showed a trend to decrease; however, they remained significantly different from baseline during the 15‐minute observation period. All interventions that involved restraint induced changes in hemodynamics and core temperature. These remained markedly elevated (*P*<0.001) throughout the restraint period without trend to decrease (Figure 3).

Changes in core body temperature (Figure 4) followed similar trends to changes in cardiovascular parameters, albeit with slower kinetics. Peak changes in core temperature were achieved several minutes following initiation of an intervention, compared with near immediate changes in cardiovascular responses. Two types of responses were observed in response to the various interventions. Moving the cage and restraint with no heating all caused a significant rise in core temperature of ≈1°C. By comparison, the effect of handling, restraint with heating with and without tail‐cuff all led to a highly significant and sustained increase in core temperature of ≈1.5 to 2°C. The presence of the investigator in the room caused a small but significant increase (*P*<0.05) when compared with the averaged baseline (Figure 4).

It is noted that when the mice were handled and restrained without heating, the initial increase in core temperature was followed by a dip and later smaller yet significant increase. This may be because of the cooling effect of the tube that was at the ambient temperature of 24±2°C and emphasizes the importance of the heating component.

**Figure 5:**
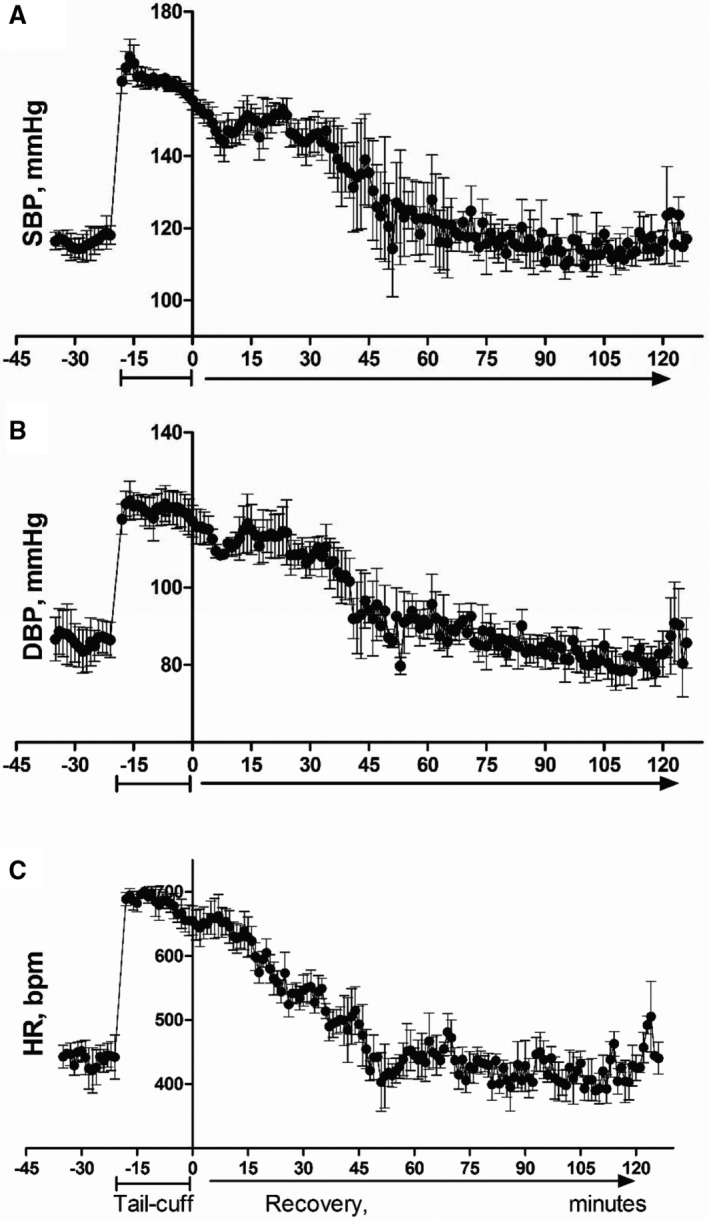
Hemodynamic profile during and until 2 hours after the tail‐cuff recording. A, Systolic blood pressure. B, Diastolic blood pressure. C, Heart rate. Values are mean±SEM for 3 animals, each data point represents 1‐minute average for 3 animals. Mice are picked up at ≈−17‐minute time point as seen on the graph. The time point “0” represents the point when the animals are released back to home cage after tail‐cuff recording is complete.

**Figure 6:**
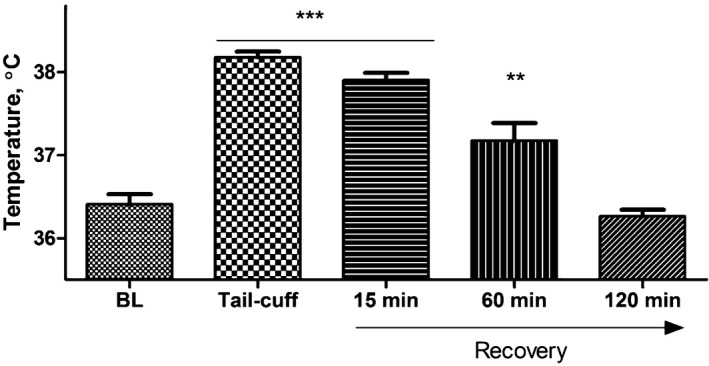
Changes in core body temperature following the tail‐cuff protocol. Values are mean±SEM for 4 mice. ***P<0.001 core temperature during the tail‐cuff and 15 minutes after this was complete vs baseline, **P<0.01 60 minutes after the tail‐cuff vs baseline (BL) repeated‐measures‐ANOVA).

Blood pressure and heart rate typically returned to baseline level 1 hour after the tail‐cuff protocol was complete and the mice were returned to home cages (Figure 5), whereas ≈2 hours were required for core temperature (Figure 6).

### Habituation (Effect of Repeated Tail‐Cuff Measurements)

**Figure 7:**
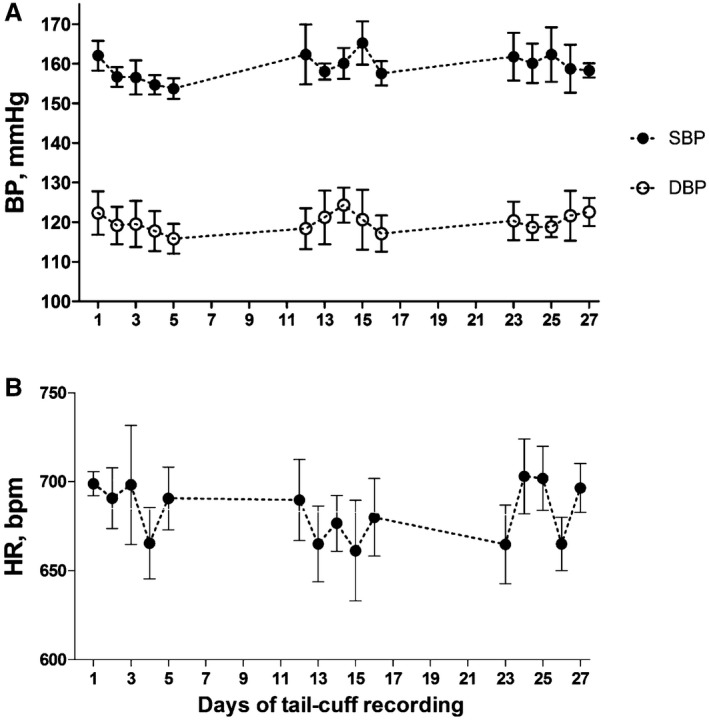
Hemodynamic telemetry recordings during repeated exposures to the tail‐cuff technique and number of accepted readings by the tail‐cuff before and after telemetry implantation. A, Systolic (SBP) and diastolic blood pressure (DBP) and (B) heart rate (HR). The results are from a study where the mice were exposed to the tail‐cuff technique on days 1 to 6, 12 to 16, and 23 to 28, where day 1 is the first day of tail‐cuff recording at least 10 days after telemetry device was implanted. The mice were rested between these days, remaining in their home cage. Values are mean±SEM for 6 mice; there was no significant difference over time as determined by the Linear Regression Analysis. bpm indicates beats per minute.

We did not observe significant changes in heart rate or blood pressure recordings as measured by telemetry when the tail‐cuff technique was carried out for 5 consecutive days over 3 periods, each period separated by 6 days rest (Figure 7). This does not support the generally accepted hypothesis that the mice develop less stress following repeated exposure to the tail‐cuff technique.

### Simultaneous and Nonsimultaneous Tail‐Cuff and Telemetry Recordings and Effect of AngII

**Figure 8:**
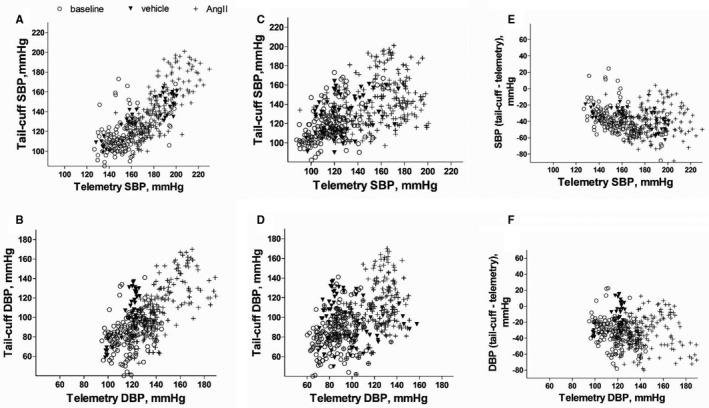
Correlation analysis of blood pressure recordings made by telemetry and tail‐cuff techniques. The correlations shown are as follows: in the same mouse simultaneously (A and B); nonsimultaneously, with telemetry readings taken before the mouse was disturbed (C and D); differences in the recordings made by tail‐cuff and telemetry simultaneous systolic (E) and diastolic (F) blood pressure vs telemetry. Data show systolic (SBP) or diastolic (DBP) blood pressure at baseline (clear circles), following vehicle infusion via minipump (filled triangles) or angiotensin II (AngII) infusion via minipump (crosses). Pearson correlations are as follows: (A) Pearson *r*=0.40, 0.88 and 0.74 at baseline, following vehicle or AngII infusion, respectively, *r*²=0.16, 0.77 and 0.55, slope=0.45±0.09, 0.70±0.07, 1.04±0.06 for each group, respectively. For the combined groups, Pearson *r*=0.76 and *r*²=0.57. B, DBP as measured simultaneously by the 2 techniques before (baseline), and after vehicle or AngII infusion: Pearson *r*=0.23, 0.88, 0.68, *r*²=0.05, 0.74, 0.46, respectively, for each group; for the combined data set, Pearson *r*=0.60, *r*²=0.44. C, SBP acquired nonsimultaneously by the 2 techniques: Pearson *r*=0.46, 0.27, 0.25, *r*²=0.21, 0.08, and 0.06 for baseline, vehicle, and AngII groups, respectively. For the combined data set, Pearson *r*=0.51; *r*²=0.26. D, DBP acquired nonsimultaneously by the 2 techniques, Pearson *r*=0.25, −0.15, 0.36, *r*²=0.06, 0.02, 0.13; for the combined data set, Pearson *r*=0.47, *r*²=0.22. E, Correlation analysis of the difference in SBP as recorded by both techniques (tail‐cuff—telemetry) vs central BP as recorded by telemetry: Pearson *r*=−0.43, −0.61 and 0.06, *r*²=0.21, 0.37, >0.01 for baseline, vehicle, and AngII groups, respectively. F, Correlation analysis of the difference in DBP as recorded by both techniques (tail‐cuff—telemetry) vs central BP as recorded by telemetry: Pearson *r*=−0.36, −0.67, and >0.01, *r*²=0.13, 0.45, >0.01 for baseline, vehicle, and AngII groups, respectively. Each data point represents 1 of the 399 pairs of simultaneous measurements (A and B), of which: baseline, 141 pairs, following vehicle, 41 pairs, or AngII infusion, 216 pairs; 437 nonsimultaneous measurements (C and D), of which: baseline, 161 pairs of recordings, following vehicle, 54 pairs, or AngII infusion, 222 pairs of data, made by telemetry and tail‐cuff in 12 mice.

**Figure 9:**
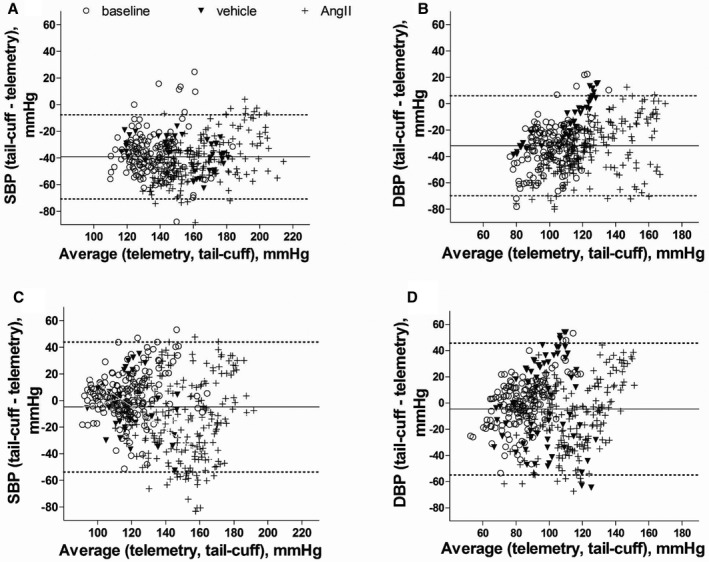
Comparison of blood pressure recordings made by telemetry and the tail‐cuff technique in the same mouse: Bland‐Altman plot of simultaneous systolic (A), diastolic (B), and nonsimultaneous systolic (C) and diastolic (D) blood pressure recordings. Systolic blood pressure (SBP) or diastolic blood pressure (DBP), as measured simultaneously or nonsimultaneously by the 2 techniques at baseline (clear circles) and following angiotensin II (AngII) (crosses) or vehicle (filled triangles) infusion. Bias±limit of agreement (±2SD) for: (A) SBP: −37.2±16.9 (at baseline), −35.8±12.0 (vehicle), and −41.2±16.4 (AngII infusion). B, DBP −33.5±18.2 (baseline), 12.3±16.9 (vehicle), and 34.4±18.8 (AngII infusion). C and D, for (C) SBP, 2.63±17.1 (baseline), 0.93±36.2 (vehicle), −15.2±29.2 (AngII infusion); and (D) DBP, −0.68±20.4 (baseline), −2.09±26.75 (vehicle), and −11.2±25.1 (AngII infusion). Each data point represents 1 of (A and B) 399 pairs of simultaneous recordings by telemetry and tail‐cuff, of which: 141 pairs or measurement by the 2 techniques were made at baseline, 41 pairs following vehicle, and 216 pairs of measurements following AngII infusion; (C and D) 437 pairs of nonsimultaneous recordings pairs, of which: 161 pairs of recordings at baseline, following vehicle—54 pairs, or AngII infusion—222 pairs of data made by telemetry and tail‐cuff in 12 mice. The solid lines represent mean difference between telemetry and tail‐cuff systems (bias) and the dotted lines show upper and lower 95% confidence limits of agreement (2 SD) for combined baseline, vehicle, and AngII groups.

A total of 399 and 437 pairs of simultaneous and nonsimultaneous recordings, respectively, obtained by both techniques with and without AngII infusion were compared (Figures 8 and 9). A medium‐strong and significant correlation (Figure 8) was found for simultaneously acquired systolic and diastolic blood pressure (SBP, DBP) when the pooled data (to include before and following AngII or vehicle infusion) were analyzed (Pearson *r*=0.7577 for SBP and 0.6033 DBP, Figure 8A and 8B). The Bland‐Altman analysis (Figure 9) of the same data revealed that the tail‐cuff readings were on average lower than the simultaneously acquired telemetry readings by 39.3±16.1 and 31.4±19.4 mm Hg (mean±SD) for systolic and diastolic blood pressure, respectively (Figure 9A and 9B).

Separate analysis of data points for SBP (Figures 8A and 9A) at baseline (before AngII infusion) revealed the following: before AngII infusion (baseline), Pearson *r*=0.40, *r* ^2^=0.17, bias=−37.2±16.9; following vehicle infusion Pearson *r*=0.88, *r* ^2^=0.77, bias=−35.8±12.0; following AngII infusion Pearson *r*=0.74, *r* ^2^=0.55, bias=−41.2±16.4. A similar trend was observed for DBP (Figures 8B and 9B), which is as follows: before AngII infusion, Pearson *r*=0.23, *r* ^2^=0.05, bias=−33.5±18.2; following vehicle infusion Pearson *r*=0.86, *r* ^2^=0.74, bias=−12.3±16.9; following AngII infusion Pearson *r*=0.68, *r* ^2^=0.46, bias=−34.4±18.8. For both SBP and DBP, correlations appear to be weaker and relationship tends to be less linear at baseline (ie, at the start of the experiments).

We speculated whether the difference between the 2 techniques increases when central blood pressure increases (Figure 8E and 8F). Interestingly, we found this was the case for mice measured at baseline, before AngII or vehicle infusion (Pearson *r*=−0.43, *r* ^2^=0.22), and following vehicle infusion (Pearson *r*=−0.61, *r* ^2^=0.37). There was no such significant relationship following AngII infusion (both Pearson *r* and *r* ^2^ are <0.01). The same findings were observed for DBP.

The nonsimultaneous recordings by telemetry and tail‐cuff show weak correlations according to the Pearson correlation analysis (*r*=0.51 for SDP and 0.47 DBP, Figure 8C and 8D). This is not surprising because, although the data points for each measuring technique were taken on the same day, they did not represent the same interval in time like the simultaneous recordings. Because the alignments of the data points are arbitrary, detailed correlation and regression analysis is not shown here. However, Bland‐Altman analysis confirmed (Figure 9C and 9D) a substantially smaller difference between the 2 techniques than for the simultaneous recordings. Tail‐cuff on average overestimated SBP by 2.7±17.5 mm Hg (bias±2 SD, mm Hg) and 0.9±26.7 mm Hg at baseline and following vehicle infusion, respectively, and underestimated SBP by 15.2±29.2 mm Hg following AngII infusion. The tail‐cuff system underestimated DBP by 0.7±20.4, 2.1±36.2, and 11.2±25.1 mm Hg at baseline and then following vehicle or AngII infusion, respectively. Additionally, we did not observe a trend that bias would remain constant by mouse or would differ between male and female mice (data not shown).

**Figure 10:**
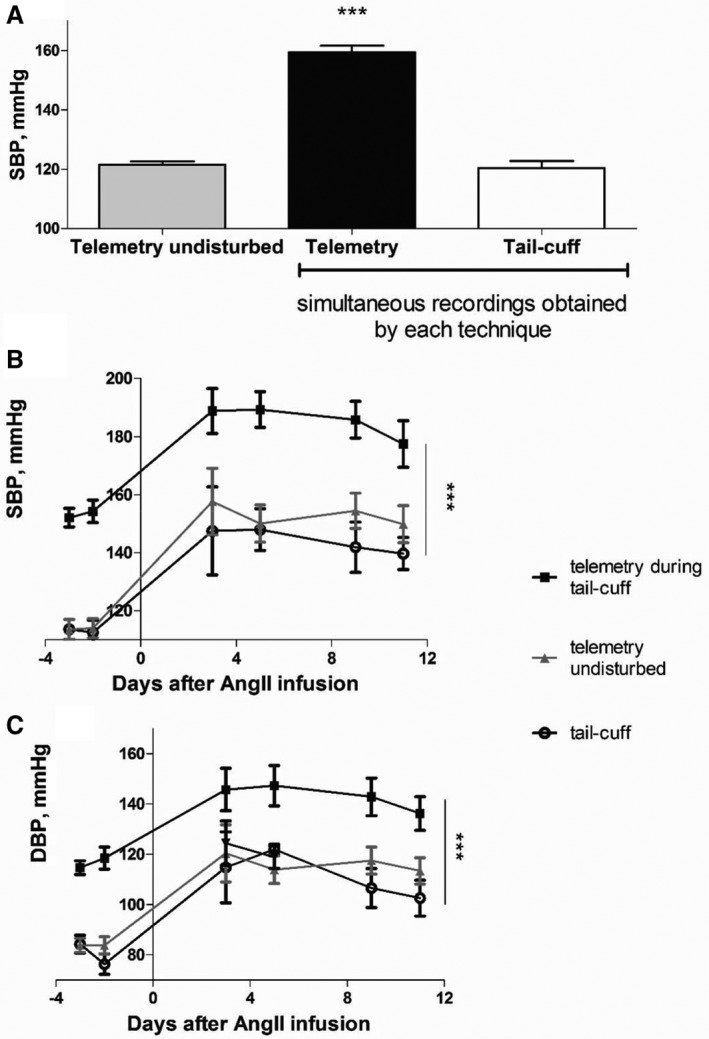
Comparison of recordings acquired simultaneously and nonsimultaneously by the 2 techniques at baseline and in the angiotensin II (AngII) hypertension model. A, SBP measured by telemetry before the animals were disturbed (gray bar) and during tail‐cuff protocol (filled bar), compared with the SBP readings obtained by the tail‐cuff technique (clear bar) in the same mouse without AngII infusion (n=6); ***P<0.001 telemetry recordings during the tail‐cuff protocol vs tail‐cuff recordings and telemetry undisturbed. B, SBP and (C) DBP determined by the 2 techniques before and following AngII infusion: measurements by telemetry before the animals were disturbed (gray triangles), during the tail‐cuff protocol (filled squares), and measurements by the tail‐cuff technique (clear circles). Values are mean±SEM for 8 mice on days −3, −2, 9, and 11, 4 mice on days 3 and 5; ***P<0.001 vs telemetry recordings during the tail‐cuff protocol repeated measures‐ANOVA). DBP indicates diastolic blood pressure; SBP, systolic blood pressure.

Systolic blood pressure can vary between individual mice and groups of mice of the same strain; however, the difference that we observed between telemetry and tail‐cuff remained. For instance, average systolic blood pressure for 6 male C57Bl/6 mice 17 to 22 weeks old equaled 120.5±9.8 mm Hg (mean±SD) by tail‐cuff versus 159.5±9.1 mm Hg by telemetry during simultaneous tail‐cuff recordings when no AngII was infused. Nonsimultaneous telemetry readings in the same mice before they were disturbed were similar to those obtained by tail‐cuff and averaged to 121.5±4.7 mm Hg (Figure 10A). By comparison, measurements by tail‐cuff and telemetry (simultaneous versus nonsimultaneous) taken in another group of C57Bl/6 mice (4 males and 4 females, 13–16 weeks old) were 113.1±10.5 for tail‐cuff and 153.2±9.3 versus 113.8±9.3 mm Hg, respectively. No difference was found between male and female mice.

A raised blood pressure was induced by AngII in male (n=4) and female (n=4 mice) mice to allow investigation of these 2 techniques under hypertensive conditions. The data for both sexes were pooled, since no differences were found between male and female mice. The raised blood pressure was detected by both techniques; a difference of ≈40 mm Hg between simultaneous telemetry and tail‐cuff recordings was maintained (Figure 10B and 10C), while the nonsimultaneous and tail‐cuff readings also remained very similar.

## Discussion

Our findings show for the first time, to our knowledge, that a range of distinct stages, typical of those associated with the tail‐cuff technique, induce large increases in central blood pressure, heart rate, and core body temperature, as measured by telemetry. Increases of blood pressure, heart rate, and body temperature are accepted markers of stress. This increase in central blood pressure and heart rate is seen, albeit to a somewhat lesser degree, merely by moving the cage that the mouse is housed in. Perhaps surprisingly, moving the cage has a similar transient effect as that of handling the mouse, with blood pressure and heart rate taking ≈15 minutes to return to baseline levels. However, an essential component of the tail‐cuff technique is the use of a holder to restrain the mouse. Our results show that restraint causes a maintained increase in both blood pressure and heart rate also following repeated exposures. It would be reasonable to conclude, based on these results, that restraint is particularly stressful to mice and may be an overriding factor in ameliorating any modifying effects of optimized handling and environmental conditions.^13^, ^32^ Moreover, it is standard to train mice so that they become accustomed to the investigator and total process; however, our results are similar to those obtained by others^23^, ^24^ in that no reduction in blood pressure or heart rate was seen as a result of repeated exposure to the technique. On the contrary, repeated restraint and tail‐cuff recording over 5 consecutive days has been used by others as a method to induce stress,^23^ in support of the concept that the restraint component of the assay is associated with hemodynamic stress. However, it is our observation that mice become familiar with both the handling and the process of tail‐cuff, which can facilitate the collection of data. The apparent stronger correlation coefficient for subsequent measuring sessions that took place later in the study supports this (Figure 8A and 8B).

It is essential to warm mice to record blood pressure using the tail‐cuff method, as recommended by the equipment manufacturer.^21^ This is achieved by using an inbuilt heating platform that also holds the restraint tubes. It is assumed that warming the animals to 33 to 36°C allows higher blood flow to the tail. The thermo‐sensitive nature of the tail means that it is otherwise constricted at the usual ambient temperature of the laboratories and animal holding rooms,^21^, ^33^, ^34^ not allowing the tail‐cuff recordings to take place. This is not surprising as ambient temperatures of the animal holding and procedure rooms have previously been shown to be below the thermo‐neutral range for mice, which is suggested to be ≈30°C.^33^ At thermo‐neutrality, mice have lower blood pressure and heart rate^33^ and vagal tone–driven control of the heart rate.^35^ Warming the heat box where the mice are placed for tail‐cuff recording to 27°C has been suggested to be a stressing factor for rodents, causing increased blood pressure and heart rate.^36^ Our results suggest that heating mice up to 35°C is not a stressing factor per se, because the unheated restrained mice show a similar blood pressure (Figure 3).

We found that body temperature was affected in a similar manner to blood pressure and heart rate. It was increased when the cage was moved and more so when handling and restraint took place. In our hands, the restraint with warming and the tail‐cuff technique were found to induce an increase in core body temperature to 38°C (approximately by 4%). However, surprisingly, when no warming was used, the initial rise in the core body temperature was attenuated. This could have been in part because of the cooling effect of the nonheated tube (maintained at room temperature) and not a sign of reduced stress, since only a reduced rise in body temperature was observed, not of blood pressure or heart rate. Although the tube's temperature was similar to the mouse's ambient temperature, contact with the tube material could interfere with the temperature homeostasis of the mouse and exert the apparent cooling effect and require a longer period in the restraint to acclimatize. Therefore, preheating the tubes to 33 to 35°C may be recommended to avoid perturbations in core temperature.

It has been previously shown that different handling techniques are associated with different levels of anxiety in laboratory animals.^13^ Hurst and West showed that when the mouse was picked up by the tail, greater anxiety is observed compared with when the mouse is lifted by a cupping technique or through use of a tube. In their study, anxiety was measured by a range of established anxiety assays.^13^ Here, we examined 3 handling techniques similar to those used by Hurst and West to determine whether any were preferable when using the tail‐cuff technique. We examined the effect of these handling techniques on blood pressure and heart rate directly using telemetry. We then carried out the various stages of the tail‐cuff technique. The results clearly show that there was little difference between the handling techniques used on central blood pressure or heart rate. We believe that this is because they preceded the standard restraint, which is associated with a high level of stress according to the increased central blood pressure and heart rate measured. Thus, none of the handling techniques tested had any immediate or downstream influence on the results obtained.

There is evidence that male researchers initiate greater stress sensations in mice.^12^ Sorge et al showed that exposure to male odors induced a significant increase in stress hormone (corticosterone) levels, whereas the response to female odor was indistinguishable from control.^12^ The same study also showed that a 15‐minute restraint induced a similar effect as the exposure to the male odor. Our data suggest that the researcher's sex makes no significant difference to the blood pressure and heart rate responses at any stage of the tail‐cuff technique.

These results show that restraint has the overriding impact on the stress of the animal, rather than the handling technique or researcher's sex. We observed ≈33% and 44% increase in blood pressure and heart rate throughout this period, respectively. Other researchers show similar changes in mice^24^ and rats.^37^, ^38^ Although other biomarkers of stress are not addressed in this study, there is a wealth of evidence on this subject,^11^, ^12^ which also support our observation that stress markers remain elevated throughout the period of restraint and show a trend to increase if the restraint period is increased.^12^

The VPR tail‐cuff system used in this study has been previously validated against telemetry,^10^ with conclusions that a negligible difference exists between the 2 techniques. We have compared 399 time‐matched recording pairs and found a very large difference between the tail‐cuff blood pressure readings and the telemetry readings, which was on average 40 mm Hg, much higher than had been previously reported.^10^ On further consideration of the only comparable study by Feng et al,^10^ they did show a similar difference between the tail‐cuff and telemetry techniques to our own within comparable ranges of central blood pressure. However, they frequently observed central systolic blood pressure <140 mm Hg (≈40% of observed values) during the tail‐cuff protocol. They showed that the tail‐cuff systematically overestimates central blood pressure when central blood pressure is <140 mm Hg as measured simultaneously by telemetry. Therefore, the difference between the 2 methods was approximately equally distributed around zero in their study. This led them and other researchers who refer to their article to suggest that there is little difference between the 2 techniques.^10^ However, in our results, we rarely observed central blood pressure <130 mm Hg during the tail‐cuff protocol, and the tail‐cuff technique reproducibly underestimated the simultaneous recordings by the telemetry.

There are differences between the protocols and mouse strains used by Feng et al and the present study, which may have given rise to the differences in the results. First, they show mixed data for C57Bl/6 and CD‐1 male mice and higher ambient temperature,^10^ whereas we used just C57Bl/6 mice. Second, they performed tail‐cuff recordings in a room heated to 25 to 30°C.^10^ Since we collected data during all stages of the tail‐cuff protocol, including the acclimatization period, we used preheated platforms and infrared lamps during the recording sessions to maintain the temperature of the mouse's tail between 33 and 35°C. Therefore, there are several reasons why a difference in the results obtained by Feng et al and this study is observed.

Other studies that use the tail‐cuff technique commonly report similar blood pressure values to those obtained by us. For example, a phenotypic screen of 37 strains of mice^22^ using the VPR tail‐cuff system (same as in this study) reported a 100±2 to 133±3 mm Hg range of SBPs for those animals. Other laboratories had comparable results using the same^39^ or different tail‐cuff systems.^40^, ^41^, ^42^ On the other hand, studies that have not concentrated on the tail‐cuff technique show elevated central blood pressure as measured by telemetry during the restraint in mice^24^, ^43^ and rats.^36^, ^37^, ^38^ Ours is the first study to show that there is a highly significant difference between simultaneous tail‐cuff and central blood pressure telemetry readings.

We realized that the nonsimultaneous telemetry blood pressure measurements (ie, those taken before the animals were disturbed for the tail‐cuff procedure) were similar to those obtained in baseline tail‐cuff readings. Moreover, both telemetry (simultaneous and nonsimultaneous recordings) and the tail‐cuff reflected the expected hypertensive response that the pressor agent AngII is associated with.^27^, ^44^ Although the nonsimultaneous and tail‐cuff readings were remarkably similar, the simultaneous readings remained significantly higher than tail‐cuff readings. This difference was observed throughout the hypertensive protocol. The relatively weaker correlation of the nonsimultaneous recordings (Figure 8C and 8D) may indicate that the tail‐cuff recordings are not a reflection of central blood pressure under stress‐free conditions, or the manner in which the nonsimultaneous samples had to be collected.

Blood pressure is a complex phenomenon resulting from anatomical and physiological characteristics of the blood vessel that contain it and those of the circulatory network that it is part of. Therefore, it is fair to expect that blood pressure readings will differ between different locations within the arterial tree and these differences might not be the same among different species. Blood pressure changes from core to the periphery in humans are characterized by pressure amplification in the resistance vessels,^45^, ^46^ whereas in the mouse, based on experimental data and in silico modeling, there appears to be gradual dampening of the pressure towards the periphery.^47^ Indeed, invasive SBP measurement from the caudal artery in an anesthetized rat shows that it is ≈17 mm Hg lower than the pressure measured from the femoral artery simultaneously.^48^ Although this technique has not been successfully performed in the mouse to our knowledge, the in silico model suggests that there is a gradual decrease in blood pressure down the abdominal aorta.^47^ It is not clear, however, whether the similarities between the resting undisturbed (nonsimultaneous) blood pressure measured by telemetry are a coincidence, or a representation of the central blood pressure that does not appear to be affected by the restraint stress.

The tail is an important thermoregulatory organ in rodents^49^, ^50^ that has both a rich blood supply and adrenergic innervation to enable efficient blood flow adjustment depending on external and internal parameters. It has been shown that the caudal artery receives between 1% and 5% of blood volume, depending on ambient temperature.^47^ These and other characteristics of this vascular bed suggest that it is markedly different from that of the conducting vessels such as the aortic arch, where telemetric blood pressure measurements often take place in the mouse, including this study.

To conclude, we found that the handling techniques and sex of the investigator have little effect on the measurement of mouse blood pressure. However, the simultaneous measurement of blood pressure by the 2 techniques reveals a remarkable divergence when simultaneous measurements are carried out. This is primarily related to the restraint step of the technique. We do not believe this has been adequately reported previously. The data generated in this quantitative study support the American Heart Association recommendations for blood pressure measurements in mice,^5^ ie, that the tail‐cuff technique can detect changes in blood pressure, similar to those observed in the AngII‐induced hypertension model here. They recommend that it be used for screening of large numbers of animals and long‐term studies where the use of the more accurate telemetry techniques has limitations, as discussed above. We believe that researchers should be aware of our findings when designing their studies that involve the tail‐cuff technique.

## Sources of Funding

This work was supported by the MRC NC3Rs (National Centre for the Replacement Refinement & Reduction of Animals in Research), British Heart Foundation, and King's College London.

## Disclosures

None.

## Supporting information


**Figure S1.** Schematic of the experimental design for the study of the effect of the handling techniques on blood pressure and heart rate. Animal groups represent different sequence of the handling techniques used. Each mouse was handled by each of the handling techniques tested in the sequence shown: A=tube handling, B=tail‐cup handling, C=tail handling. Six mice were used in this study; the results are shown in Figure 1.
**Figure S2.** A schematic to illustrate the additive approach to investigate the effect of the following factors and steps that are associated with obtaining tail‐cuff measurements: presence of the researcher in the room, moving the mouse in the cage close to the equipment, handling to place the mouse in the restraint tube, heating, and finally measuring blood pressure by the tail‐cuff. Typically, each mouse was subjected to interventions 1 to 6 on at least 2 occasions on separate days. The results are shown in Figures 3 and 4.
