# Supplementary material for: Tail‐Cuff Technique and Its Influence on Central Blood Pressure in the Mouse
Source: J Am Heart Assoc. 2017 Jun 27;6(6):e005204. doi: 10.1161/JAHA.116.005204 (PMC5669161; doi:10.1161/JAHA.116.005204)

SUPPLEMENTAL MATERIAL

Figure S1. Schematic of the experimental design for the study of the effect of the handling techniques on blood pressure and heart rate. Animal groups represent different sequence of the handling techniques used. Each mouse was handled by each of the handling techniques tested in the sequence shown: **A**=tube handling, **B**=tail-cup handling, **C**=tail handling. Six mice were used in this study, the results are shown in Figure 1.

<table>
<tr><th rowspan="4">Mice trained to the tail-cuff technique</th><th rowspan="4">Telemetry surgery + 10 day recovery</th><th>Animal groups</th><th>Handling Week 1</th><th rowspan="4">Rest, 6 days</th><th>Handling Week 2</th><th rowspan="4">Rest, 6 days</th><th>Handling Week 3</th></tr>
<tr><td>ABC</td><td>A</td><td>B</td><td>C</td></tr>
<tr><td>BCA</td><td>B</td><td>C</td><td>A</td></tr>
<tr><td>CAB</td><td>C</td><td>A</td><td>B</td></tr>
</table>

Figure S2. A schematic to illustrate the additive approach to investigate the effect of the following factors and steps are associated with obtaining tail-cuff measurements: presence of the researcher in the room, moving the mouse in the cage close to the equipment, handling to place the mouse in the restraint tube, heating and finally measuring blood pressure by the tail-cuff. Typically, each mouse was subjected to interventions 1 – 6 on at least 2 occasions on separate days. The results are shown in Figures 3 and 4.

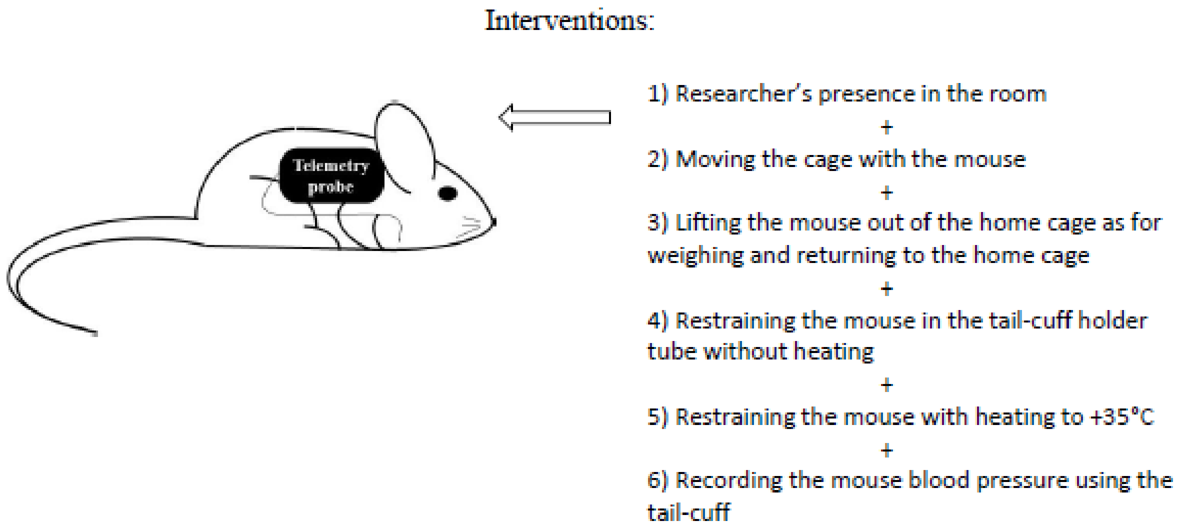

Supplement: Supplementary file 1 — Figure S1. Schematic of the experimental design for the study of the effect of the handling techniques on blood pressure and heart rate. Animal groups represent different sequence of the handling techniques used. Each mouse was handled by each of the handling techniques tested in the sequence shown: A=tube handling, B=tail‐cup handling, C=tail handling. Six mice were used in this study; the results are shown in Figure 1. Figure S2. A schematic to illustrate the additive approach to investigate the effect of the following factors and steps that are associated with obtaining tail‐cuff measurements: presence of the researcher in the room, moving the mouse in the cage close to the equipment, handling to place the mouse in the restraint tube, heating, and finally measuring blood pressure by the tail‐cuff. Typically, each mouse was subjected to interventions 1 to 6 on at least 2 occasions on separate days. The results are shown in Figures 3 and 4. [file JAH3-6-e005204-s001.pdf]
